# Genetic diversity, asexual reproduction and conservation of the edible fruit tree *Spondias purpurea* L. (Anacardiaceae) in the Costa Rican tropical dry forest

**DOI:** 10.1371/journal.pone.0277439

**Published:** 2022-11-17

**Authors:** E. Jacob Cristóbal-Pérez, Eric J. Fuchs, Jorge Lobo, Mauricio Quesada

**Affiliations:** 1 Instituto de Investigaciones en Ecosistemas y Sustentabilidad, Universidad Nacional Autónoma de México, Morelia, Michoacán, México; 2 Laboratorio Binacional de Análisis y Síntesis Ecológica, UNAM-UCR, México-Costa Rica; 3 Centro de Investigación en Biodiversidad y Ecología Tropical, Universidad de Costa Rica, San José, Costa Rica; 4 Laboratorio Nacional de Análisis y Síntesis Ecológica, Escuela Nacional de Estudios Superiores Unidad Morelia, Universidad Nacional Autónoma de México, Morelia, Michoacán, México; 5 Escuela de Biología, Universidad de Costa Rica, San José, Costa Rica; Central University of Punjab, INDIA

## Abstract

The term *circa situm* has been used to describe different conservation strategies within agricultural landscapes. *Circa situm* conserves planted or remnant species in farmlands, where natural vegetation has been modified through anthropogenic intervention. It has been proposed that trees planted or retained under *circa situm* conditions may contribute to maintaining genetic diversity, however information on the role of this strategy in preserving genetic diversity is scarce. The aim of this study was to determine the levels of genetic diversity and structure, and mating patterns in planted and unmanaged stands of the tropical fruit tree *Spondias purpurea* L. in north western Costa Rica. In three localities, we used seven polymorphic microsatellite loci and genotyped 201 adults and 648 seeds from planted and wild stands. We found no differences in genetic diversity among planted and wild stands. Genetic structure analysis revealed that gene flow occurs among planted and wild stands within localities. Clones were present and their diversity and evenness were both high and similar between planted and wild stands. The number of pollen donors per progeny array was low (N_ep_ = 1.01) which resulted in high levels of correlated paternity (r_p_ = 0.9). Asexual seeds were found in 4.6% of the progeny arrays, which had multilocus genotypes that were identical to the maternal trees. Our results show that although planted stands under *circa situm* conditions can maintain similar levels of genetic diversity than wild stands, the low number of sires and asexual seed formation could threaten the long term persistence of populations.

## 1. Introduction

Tropical dry forests (TDFs) constitute one of the most important reservoirs of biodiversity in the tropics, with high levels of species richness and endemism [1–5]. TDFs are also socially and economically important because a considerable number of species found in TDFs are used by humans for food, firewood, fodder, medicine, construction, live fencing, ornaments and crafts, rituals, and leather tanning [6–8]. This utilitarian value of TDFs as sources of timber and non-timber products has been widely recognized as an argument for their conservation and sustainable management [9–11]. However, despite the high biodiversity of TDFs, they are one of the most threatened ecosystems in the tropics [12, 13], with less than 10% of their original extent remaining [13, 14]. Human preference for the seasonally dry tropical environment [15], the ease of clearing its vegetation and suppress future regeneration with fire [3], have led to the destruction and fragmentation of this habitat [16].

In north western Costa Rica, TDFs have been reduced to a series of small forest patches surrounded by large cattle fields or cultivated areas (e.g., sugar cane and rice fields). These forest patches shelter only 0.1% of the original TDF cover in Costa Rica [3, 17]. Several studies have demonstrated that habitat loss and fragmentation negatively affect the reproductive success and genetic diversity of tropical trees, compromising the long term survivorship of populations [18–22]. However, isolated trees that are maintained by farmers, can serve as “stepping stones” between forest patches and, as a result, play a critical role in gene movement and connectivity among tropical forest fragments [23], significantly contributing to the propagule pool of remnant forests [24]. In addition, trees growing among farmlands and in neighboring natural forest fragments may also play an important role maintaining populations of insects, birds and mammals needed for crop pollination, biological pest control, and increasing crop productivity [25–27]. In agricultural landscapes, this arrangement of trees growing within farmlands and in remnant forest fragments, allow farmers to maintain crop production while indirectly conserving species richness and genetic diversity in what can be interpreted as *circa situm* conservation [28].

The term *circa situm* has been used to describe different conservation strategies within agricultural landscapes (e.g., agroforestry systems, home gardens, living fences, urban amenities) outside natural habitats, but within the native range of the species [16]. Generally, *circa situm* conserves planted or remnant trees in farmlands or forest patches where natural forests or woodlands containing the same trees were once found; but where natural vegetation has been lost or modified significantly through anthropogenic intervention [28]. *Circa situm* plantations are not normally created for conservation [16], but rather tree stands are planted to provide resources such as food, fibers, medicine, live fences, and edibles among others [28]; or more recently to provide amenities and comfort in urban parks and streets [29]. Indirectly, they allow for the conservation of different organisms. However, *circa situm* plantations need to be sufficiently close to wild populations, so that pollen flow (i.e., gene flow) from wild individuals into planted stands is still possible, securing fruit production and reducing the loss of genetic diversity [28, 30–33].

*Spondias purpurea* L. is a dioecious fruit tree domesticated in the Mesoamerican region [34, 35] known locally as “jocote”, “ciruelo” or “abal”. Jocotes were domesticated for their plumlike fruits, which are collected from rustic plantings and sold fresh in local markets or made into jams and beverages [34, 36, 37]. *Spondias purpurea* is pollinated by social bees and wasps and fruits are dispersed by small mammals and birds [22]. During fructification, wild *S*. *purpurea* fruits are easily distinguished by their fruits which are usually smaller, more acidic and less fleshy than cultivated fruits [35, 38]. In Mesoamerica, *S*. *purpurea* like other perennial domesticated species is generally vegetatively propagated by stem cuttings in homegardens and living fences, frequently in close proximity to wild stands (unmanaged trees), that occur in secondary forests and TDF’s remnants [35]. Natural populations of this fruit tree face the negative reproductive and genetic consequences of TDF loss and fragmentation [22], however information on the role of cultivated trees in the maintenance of its genetic diversity is lacking. In other species, research has shown that cultivated individuals can help maintain genetic diversity in managed landscapes [30–32]. Female *S*. *purpurea* trees are more likely to be vegetatively propagated in *circa situm* and along live fences for fruit consumption. Fruit production can be achieved via sexual reproduction, if male trees from nearby populations act as pollen sources or via asexual reproduction (i.e., apomixis) [37]. Planted stands, predominantly composed of female trees, receive less pollen from fewer donors, because male *S*. *purpurea* flowers offer pollen and nectar, while female flowers only offer nectar as a reward, thus females are less likely to attract pollinators [22]. This may result in a higher proportion of asexual fruit production in planted stands [37], and fruits produced via sexual reproduction, are expected to have a lower number of sires compared to fruits from wild stands.

Our study took place in the north west pacific region of Costa Rica, where wild stands of *S*. *purpurea* in secondary forests and isolated trees are in close proximity to plantings along live fences, small farms or in homegardens. The objectives of our study were (1) to compare genetic diversity and structure among wild and planted stands of *S*. *purpurea* (2) to estimate mating patterns in terms of correlated paternity of wild and planted stands of *S*. *purpurea*, and (3) to determine the frequency of sexual and asexual seeds produced in wild and planted stands of *S*. *purpurea*. This will allow us to analyze the role of planted stands for *circa situm* conservation of genetic diversity in this tropical dioecious species.

## 2. Methods

### 2.1 Study area and sampling

**Fig 1:**
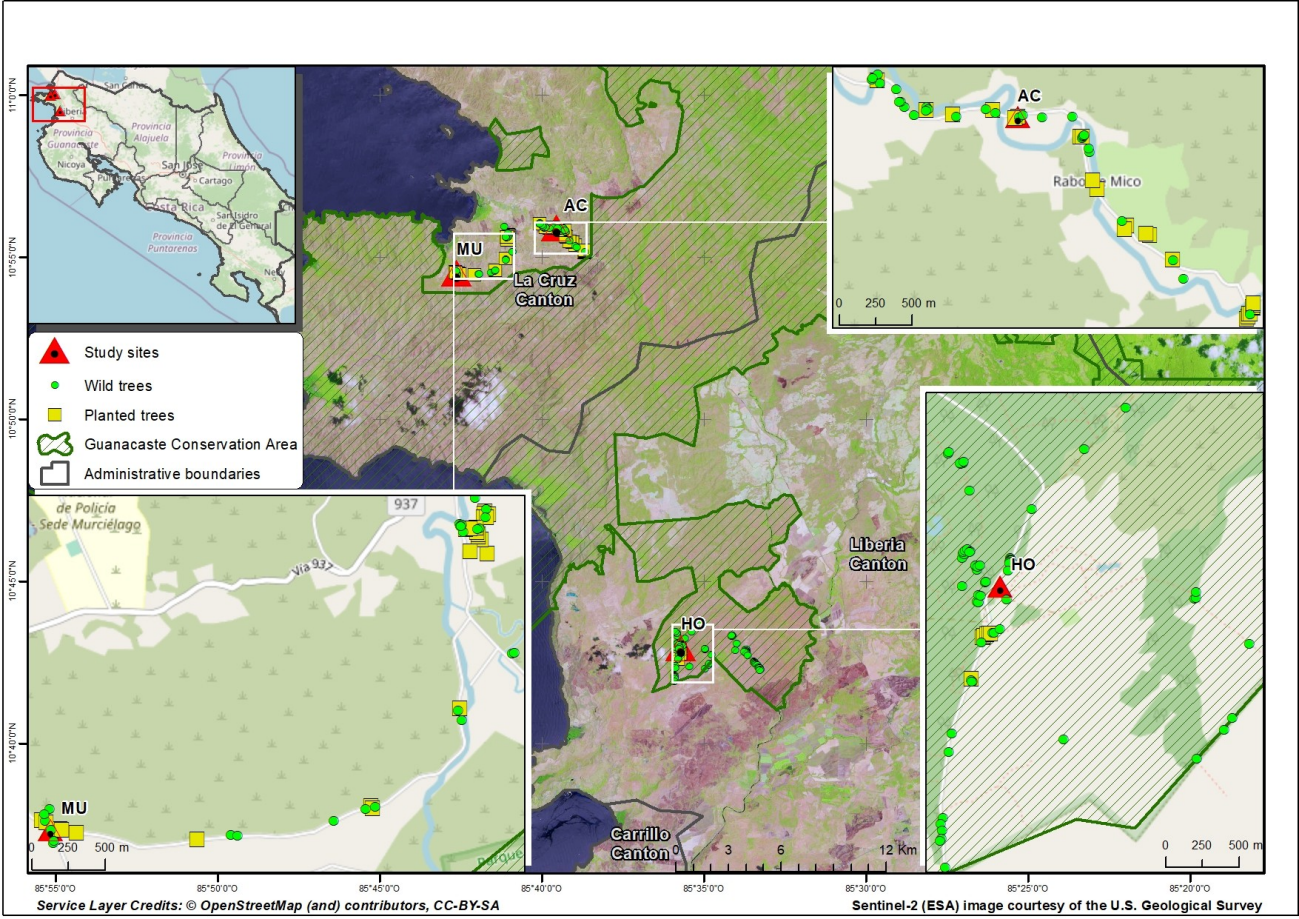
Spatial distribution of planted and wild *Spondias purpurea* individuals in three study sites: A) Agua Caliente (AC), B) Murciélago (MU) and C) Horizontes Forestal Research Station (HO), in the North western region of Costa Rica. This map was generated with data from OpenStreetMap.

The study was conducted in north western Costa Rica in the province of Guanacaste. The study region consists primarily of TDF with a mean annual rainfall of 1600 mm and a marked dry season that extends from December to May [21]. The majority of the TDF in the study area was destroyed by the timber and cattle industry in the second half of the twentieth century, and has been converted to pastures and agricultural fields [17]. Presently, most *S*. *purpurea* individuals may be found in rustic plantations, propagated vegetatively for fruit consumption or found in natural habitats. Planted and wild stands of *S*. *purpurea* were located in three localities: Agua Caliente (AC), Murciélago (MU) and Horizontes Forestal Research Station (HO) (Fig 1). The three study sites are all TDFs that differ in their land use. Agua Caliente (AC) and Murciélago (MU) are disturbed habitats mainly composed of remnant forest patches and isolated trees surrounded by an agricultural matrix (Fig 1). Along both sites planted trees are found in backyard gardens and living fences, while wild trees grow within small forest remnants and in pasture lands as isolated trees (Fig 1). HO is a 7317 ha managed secondary forest. In the past, large portions of HO were used for rice, cotton, and sorghum production, as well as cattle grazing. However, over three decades ago the site was converted to an experimental station and was allowed to regenerate [39, 40]. At HO planted stands are trees vegetatively propagated in backyard gardens near station facilities, wild trees are growing in a forest remnant along a brook and in secondary forests that are part of the natural regeneration program of the station (Fig 1). Within each study site, we located, marked and mapped all adult *S*. *purpurea* trees. Sex ratio per site was expressed as the proportion of males in the population: males / (females + males) [41]. To determine deviations from 1:1 sex ratio we performed a goodness of fit G-test for all sites using the *stats* library implemented in the R [42]. We collected fresh leaf tissue from adults (male and female trees) and stored them at -20° C until DNA extraction. To estimate mating patterns and correlated paternity of the progeny, we collected 15 fruits per female tree, directly from the canopy of 20 female trees both in planted and wild stands. We dissected fruits and sampled diploid embryos for DNA extraction. The permits to access and collect within the protected areas of the study were granted by the Research Program of the Área de Conservación Guanacaste (AGC) to the researchers of this study (Permit number: ACG-023-2018).

### 2.3 DNA extraction and microsatellite amplification

DNA from leaves and embryos was extracted using a modified Cetylmethylammonium Bromide (CTAB) protocol [43]. Ten microsatellites previously developed for *S*. *purpurea* [44], were amplified via multiplex PCR using QIAGEN Multiplex kit (QIAGEN, Hilden, Germany) in 12 μL reaction volumes. Multiplex PCR amplification conditions followed Cristóbal-Pérez et al., [38] and fragments were analyzed on an automatic ABI-PRISM 3100-Avant sequencer (APPLIED BIOSYSTEMS, Carlsbad, California, USA), using GeneScan LIZ 600 (APPLIED BIOSYSTEMS) to determine fragment sizes. Alleles were scored manually using *GeneMarker Software* version 2.6.4 (SOFTGENETICS). To reduce genotyping error, all samples were independently scored by two different researchers to reach a consensus in the final data set. In addition the software MICROCHECKER [45] was used to detect the presence of null alleles and large allelic dropout across all loci. Three loci (SPUR41, SPUR35 and SPUR39) that were monomorphic in all populations were excluded from further analyses.

### 2.4 Genetic data analysis

A total of 201 adult individuals (46 planted and 155 wild) and 600 seeds (281 planted and 319 wild) were sampled and genotyped in the three study sites. Genetic diversity was quantified for planted and wild stands using the following parameters: allele number averaged across loci (*Na*), allelic richness (*Ar*), observed (*Ho*) and expected heterozygosities (*He*), and inbreeding coefficients (*F*). Allelic richness was estimated by rarefaction of alleles as implemented in the *hierfstat* library [46]. All other diversity parameters were calculated using the library *poppr* [47] implemented in R [42]. All parameters were calculated using the complete data set and a data set excluding repeated matching multilocus genotypes (i.e., clones). Differences in genetic diversity parameters (H_e_, H_o_ and F) between planted and wild stands were analyzed using 10, 000 permutations to test significance in *GenoDive v*.*3*.*04* [48]. A permutation test was also used to test if inbreeding coefficients differed significantly from zero. We used *GenAlEx v*.*6*.*5* [49, 50] to estimate the probability of identity (PI), which estimates the probability of randomly sampling identical genotypes. In order to identify individuals that share the same multilocus genotypes (genets) we used the software GENODIVE v.3.04 [48]. Individuals with the same multilocus genotypes were assigned to the same *genet*. Genotypes with missing data were ignored [48]. After we calculated the number of unique multilocus genotypes, Simpson’s genotypic diversity and genotypic evenness indexes were estimated. The Simpson genotypic index [48], was calculated as:

$$D=1-\sum p_{i}^{2}$$

where *p*_*i*_ are allele frequencies. This index provides an estimation of the probability that two randomly selected genotypes are different and scales from 0 (no genotypes are different) to 1 (all genotypes are different). Genotypic evenness is a measure of the distribution of genotype abundances, which takes values from 0 to 1 [48]. A genotypic evenness of 1 indicates that in a population all genotypes are equally abundant; while an evenness closer to zero is expected for a population dominated by a single unique genotype [48]. The genotype diversity was estimated by using R = G − 1/N − 1, where G is the number of distinct genotypes identified and N is the number of shoots analyzed; R will always be zero for a single clone stand and one for maximal genotypic diversity, when every sampled unit is a new genet [51].

To explore the overall genetic structure of planted and wild stands, we used the software STRUCTURE [52, 53] to determine the best configuration of samples into K clusters based on similarity in allele frequencies, and possible admixture among clusters. We used the admixture model with correlated allele frequencies, with 250 000 MCMC chains and a burning of 25 000 chains. We estimated the likelihood of each configuration for K between 2 and 8, using 15 replicates for each K value. StructureHarvester v0.6.94 [54] was used to determine the most likely number of K clusters, and the Q-matrices were merged using the Full-Search Algorithm implemented in CLUMPP 1.1 [55]. CLUMPP’s output was visualized using the popHelper library [56]. We estimated differences in allele frequencies among planted and wild stands using Nei’s G_ST_ statistic [57], with 1000 permutations to test for significance using *GenoDive v*.*3*.*04*. We estimated differences using all the genotyped individuals, and also on a reduced dataset with a single individual per clone. Relatedness of individual trees was estimated for the planted and wild stands using the Loiselle’s kinship coefficient (F_IJ_) [58], estimated in *GenoDive v*.*3*.*04*. This coefficient is based on the relative probability of identity by descent of the alleles within the two compared individuals [58].

Multilocus correlation of paternity (*r*_*pm*_) was estimated using MLTR [59]. Correlated paternity is a measure of the proportion of pairs of outcrossed siblings that are full siblings. The standard error of the estimates was calculated by bootstrapping with 1 000 repetitions. We estimate the average effective number of pollen donors per maternal plant (N_*ep*_) as the reciprocal of the *r*_*pm*_ [59].

## 3. Results

We did not find statistical differences between planted and wild individuals for observed and expected heterozygosities, nor for inbreeding coefficients (Table 1, S1 and S3 Tables in S1 Data). MICROCHEKER did not find any evidence of null alleles, nor allelic dropout for all analyzed loci. The probability of identity (PI) estimated using all seven loci was *PI* = 0.00003. The proportion of distinguishable genets based on multilocus genotypes in planted trees (R = 0.74) was similar to wild trees (R = 0.77) (Table 2). Clonal diversity was high and similar between planted (D = 0.90) and wild trees (D = 0.96). Clonal evenness measures were also similar between planted (E = 0.70) and wild trees (E = 0.62) (Table 2). Genetic diversity estimates did not deviate significantly from our initial estimates, when clones were removed (S2 Table in S1 Data). Sex ratios were female biased (G = 12.71, p < 0.001) for AC and MU, but did not deviate significantly from 1:1 ratios in HO (S4 Table in S1 Data) (p>0.05).

**Table 1:** Genetic diversity parameters of wild and planted stands of *Spondias purpurea* in northwestern Costa Rica, using seven microsatellite loci developed by Cristobal-Perez et al. (2019).

Group	Size Class	N	N_a_	*A*_*r*_	H_o_	H_e_	F
Planted	Adults	46	3.33	2.92	0.545	0.497	-0.031
			(0.986)	(0.11)	(0.162)	(0.144)	(0.073)
	Seeds	281	3.95	2.07	0.580	0.474	-0.217
			(0.406)	(0.031)	(0.097)	(0.015)	0.160
	**Mean**		**2.38**	**1.68**	**0.431**	**0.362**	**-0.45**
Wild	Adults	155	4.19	2.85	0.512	0.489	0.010
			(0.423)	(0.134)	(0.089)	(0.042)	(0.122)
	Seeds	319	3.95	2.138	0.557	0.477	-0.157
			(0.238)	(0.152)	(0.061)	(0.036)	(0.04)
	**Mean**		**4.071**	**2.157**	**0.535**	**0.483**	**-0.074**

N: number of individuals; N_A_: mean allele number per locus (±SD: standard deviation); A_R_: allelic richness (±SD); H_O_: observed heterozygosity (±SD); H_E_: expected heterozygosity (±SD); F: inbreeding coefficient (±SD).

**Table 2:** Clonal diversity parameters of adult trees in wild and planted stands of *Spondias purpurea*.

Group	Locality	N	*G*	*R*	*D*	E
Planted	AC	19	12	0.61	0.9	0.56
	MU	11	7	0.60	0.81	0.55
	HO	7	7	1	1	1
	**Mean**		**8.66**	**0.74**	**0.9**	**0.7**
Wild	AC	28	21	0.74	0.94	0.51
	MU	20	19	0.95	0.99	0.95
	HO	84	53	0.63	0.96	0.41
	**Mean**		**31**	**0.77**	**0.96**	**0.62**

N: Number of individuals; G: number of unique multilocus genotypes; R: Genotype diversity; D: Simpson’s Diversity; E: evenness. AC: Agua Caliente; MU: Murciélago; HO: Horizontes Forestal Research Station.

**Fig 2:**
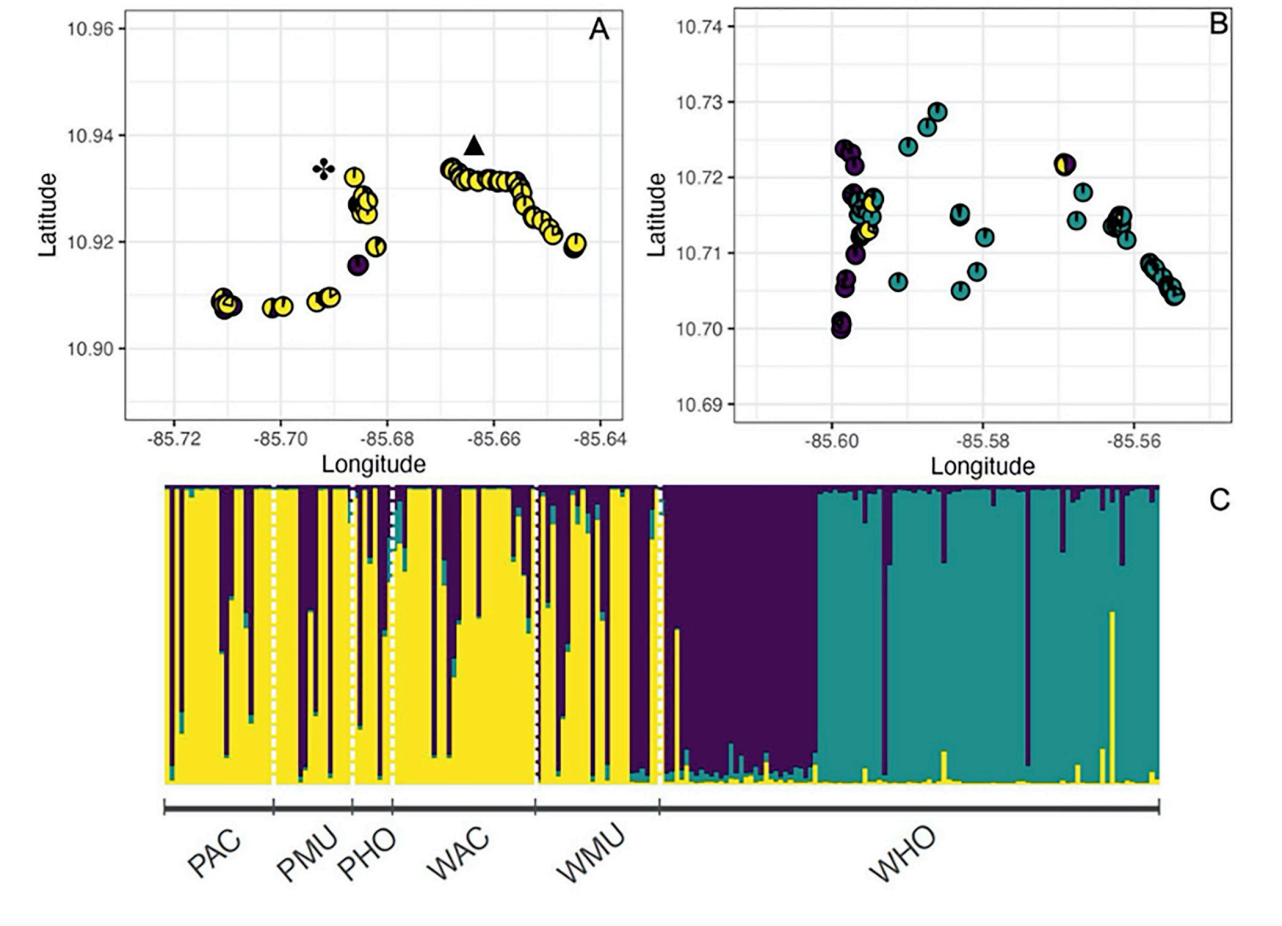
Geographic location of sampled trees within study sites. A) location of trees in Agua Caliente (AC: ✤) and Murciélago (MU: ▲). B) Location of sampled trees and in Horizontes (HO). Pies represent the percentage of each individual cluster assignment to one of three clusters as indicated by STRUCTURE. C) Bayesian assignment performed by STRUCTURE among planted and wild stands of *Spondias purpurea* in north western Costa Rican for K = 3 clusters. Each vertical bar represents an individual and is divided proportionally to the probability of assignment of each individual to each genetic cluster. Colors in panels A and B are the same as those in panel C. Letters P and W before study site abbreviation indicate Planted and Wild stands, respectively.

StructureHarvester suggested that K = 3 clusters was the most likely configuration for all adult individuals. When individuals were grouped into K = 3 clusters, the observed division did not fully correlate with the division into wild and planted stands. Individuals in three planted (PAC, PMU, PHO) and two wild stands (WAC, WMU) are predominantly grouped into cluster 1, with evidence of admixed individuals from cluster 2 (Fig 2C). WHO individuals are grouped by STRUCTURE into two separate clusters (Fig 2C). In addition, pairwise G_ST_ estimates show a lack of differences in allele frequencies between planted and wild stands within sites, with the exception of HO (S5 Table in S1 Data). All wild populations differed in allele frequencies among them (S5 Table in S1 Data), while planted stands had a higher level of similarity among them. Genetic structure patterns were similar when clones were removed from the analysis (S6 Table in S1 Data).

Paternity correlations were similar for both planted and wild progenies (*r*_*p PLANTED*_ = 0.99; *r*_*p WILD*_ = 0.91) (Table 3). Therefore, the effective number of pollen donors was approximately one for both planted and wild trees (*N*_*ep*_ = 1.01; 1.09 respectively). We identified 30 seeds with multilocus genotypes that were identical to the maternal tree (MGM), which suggests asexual seeds production (Table 4). Our kinship analysis (S7 Table in S1 Data) shows that in almost all cases, trees from within stands show elevated kinship values (diagonal of S7 Table in S1 Data). Lower kinship estimates are observed when cultivated or planted trees are compared among sites. Planted and wild trees within sites also show elevated kinship values, with the exception of WHO (Wild trees at HO) and PHO (planted trees at HO) that have a pairwise kinship of *F*_*IJ*_ = 0.012 (S7 Table in S1 Data).

**Table 3:** Paternity correlation of wild and planted stands of *Spondias purpurea*.

Tree type	Locality	n	*r*_*p*_	*Nep*
**Planted**	AC	141	0.99 (0.03)	1.01
	MU	154	0.99 (0.01)	1.01
	EH	116	0.99 (0.09)	1.01
	**Mean**	**411**	**0.99**	**1.01**
**Wild**	AC	84	0.95 (0.06)	1.05
	MU	47	0.83 (0.07)	1.2
	EH	107	0.95 (0.04)	1.05
	**Mean**	**238**	**0.91**	**1.09**

n: offspring; *r*_*p*_: multilocus correlation of outcrossed paternity; *Nep*: effective number of parents. Standard errors are given in parentheses. AC: Agua Caliente; MU: Murciélago; HO: Horizontes Forestal Research Station.

**Table 4:** Progeny arrays with multilocus identical genotypes to maternal trees, from wild and planted trees of *Spondias purpurea*.

Tree type	Locality	Maternal tree	*n*	*Mig*
**Planted**	AC	ACF-16	10	5
		ACF-38	16	5
	MU	MUF-41	10	5
		MUF-3	16	5
	EH	HF-1	13	1
**Wild**	MU	MUF-24	17	7
		MUF-49	12	2

*n*: number of seeds analyzed per tree; *Mig*: number of seeds with multilocus genotypes identical to maternal tree. AC: Agua Caliente; MU: Murciélago; HO: Horizontes Forestal Research Station.

## 4. Discussion

Genetic diversity estimates in this study were comparable to those found in other tropical trees [60–62] and species of the genus *Spondias* [63–65]. The comparison of genetic diversity parameters between *S*. *purpurea* planted and wild stands showed no differences, suggesting that *circa situm* conditions may have conserved genetic diversity in adults. Our genetic diversity estimates were similar to those found in natural and fragmented Mexican populations of *S*. *purpurea*, and comparable to genetic diversity values found in other perennial edible plants (e.g., *Carya illinoinensis* (Wangenh.) K. Koch., *Malus domestica* (Suckow) Borkh., *Olea europea* L., *Pistacia vera* L., *Prunus avium* L., *Castanea dentata* (Marsh.) Borkh., *Vitis vinifera* L., *Leucaena esculenta* (DC.) Benth., *Polaskia chichipe* (Rol-Goss.) A.C.Gibson & K.E.Horak, *Euterpe edulis* Mart.) [33, 37]. In all these previous cases, planted stands had comparable levels of genetic diversity to their wild relatives [37], which is contrary to expectations that domesticated species should have lower genetic diversity than their wild counterparts [66].

Our results show the presence of trees with identical multilocus genotypes (i.e., clonal individuals) both in planted and wild stands. In addition, a small proportion of progenies have multilocus genotypes that are identical to their maternal trees, which is an indirect evidence of apomictic seed formation [67, 68]. Clonal diversity was similarly high in both planted and wild tree stands (Table 2). High clonal diversity in planted stands can be explained as a result of clonal propagation, which is commonplace in vegetatively propagated plants (e.g. *Ficus carica* L., *Dioscorea rotundata* Poir., *Olea europaea* L., *Theobroma cacao* L.*)* [69–72]. At our study sites, clonal genotypes in wild stands are scattered trees in secondary forests, probably established as a result of dispersion of asexually produced seeds. Some cultivated perennial plants have evolved from producing fruit through sexual reproduction in the wild, to parthenocarpic fruit production under different management intensities (e.g., *Musa paradisiaca* L., *Ficus carica* L., *Pyrus communis* L., *Pistacia vera* L.) [37]. This reproductive mechanism can be especially important in fragmented, human-disturbed habitats, where lower mating probabilities and changes in pollinator assemblages are common, increasing the uncertainty of animal pollination [22, 73]. However, apomictic seeds may reduce the genetic diversity of future generations, particularly for species with reduced effective population sizes [74, 75]. Thus, seed production in *circa situm* stands may not be a viable option as propagule sources for regeneration or the creation of future orchards.

The genetic admixture observed in individuals of wild and planted stands at AC and MU suggests that planted trees in these sites were likely selected from seedlings or cuttings of trees in nearby wild populations. In contrast, at HO the genetic differences observed between wild (WHO) and planted (PHO) stands reflect that planted trees (PHO) may have been propagated from other locations with allele frequencies similar to those observed at AC and MU. Planted trees are commonly selected for their larger fruits, and our results suggest that these trees may have been selected from a few sources or farmers have propagated similar genotypes across all planted stands. During the process of domestication trees that bear large, fleshy, sweet fruits have been selected, and also those that can be reproduced easily from cuttings [35]. Our results indicate that planted individuals of *S*. *purpurea* may originate from a few genotypes that have become common in planted and wild stands in Guanacaste. Both our STRUCTURE and G_ST_ results suggest that gene flow is likely to occur between wild and planted stands within sites. Furthermore, individual STRUCTURE assignments present indirect evidence of gene flow among sites; however this occurs less frequently, as evidenced by significant pairwise G_ST_ estimates among populations. In the agro-ecosystems we studied, the source of the planted populations may have been nearby wild populations, which would explain the comparable levels of genetic diversity and the low levels of genetic differentiation observed between wild and cultivated plantings. In our study, planted stands are small orchards and live fences in close spatial proximity to wild populations, which inhabit nearby secondary forests or forest remnants. The fact that planted trees likely originated from nearby wild populations and the unconstrained gene flow among planted and wild trees, is congruent with the observed levels of genetic structure among wild and planted populations. There is evidence that gene flow can be sustained between planted plants and their wild relatives by pollinators inhabiting forest remnants [72, 76]. An interesting result is the genetic structure observed between individuals in the wild trees at HO (WHO, Fig 2B and 2C). The purple cluster corresponds to individuals found at a forest remnant along a brook in a mature forest area that has never undergone management. In contrast, individuals assigned by STRUCTURE to the teal cluster were collected in a forest patch that has regenerated recently (~ 30–40 yrs) [39, 40]. The second site represents an area under passive ecological restoration in the ACG, where fires and non-native pastures are controlled to allow for natural tree regeneration [77]. These differences in the history of both sites at HO may represent different colonization or founder effects, resulting in differences in allele frequencies that were picked up by the STRUCTURE algorithm. This underlying structure at HO also influences the pairwise G_ST_ values that show that HO differs significantly from all other populations, supporting our previous suggestion that trees at AC and MU probably have different origins compared to HO. Therefore, population historical origin may have a greater influence on genetic diversity and better explain the structure than the simple comparison between planted and wild stands.

Previous studies have shown that *S*. *purpurea* depends on pollinators for sexual reproduction and seed production [22, 38]. In this study, paternity correlations indicated that seeds from both planted and wild trees were sired by a low number of effective pollen donors and asexual reproduction. We also observed elevated pairwise kinship values within sites, which can increase the relatedness of progeny arrays. In dioecious plants, a reduced number of sires can be related to lower mate availability (e.g. skewed sexual ratios) and changes in pollinator behavior [78–82]. High correlated paternity estimates at AC and MU can be explained by a lower mate availability caused by female biased sex ratios (male/female <0.26). The scarcity of males may lead to the overrepresentation of a few pollen donors in the progeny [24], increasing the relatedness of seeds. The large correlated paternity observed in the progeny of *S*. *purpurea* could also be related to pollinator behavior. In *S*. *purpurea*, a previous study showed that in disturbed habitats, floral displays are larger but pollinator visitation is negatively affected, reducing fruit-set and increasing paternity correlation [22]. Larger floral displays reduced pollinator movement between reproductive individuals, reducing the diversity of sires and increasing the relatedness of maternal progeny arrays [21, 22, 82–84]. Cristóbal-Pérez et al. [22] showed that in fragmented habitats in Mexico, paternity correlation was high (r_p_ = 0.63) as a result of a lower number of sires (N_ep_ = 1.58). Our results show a limited number of sires both in planted and wild stands of *S*. *purpurea* in Costa Rica. Pollinator behavior may explain the high correlated paternity observed in the progenies at HO, where sex ratios did not statistically differ from a 1:1 ratio. These results are congruent with previous findings that habitat loss and fragmentation reduce the number of pollen donors and progeny fitness [20–22, 83]. Planted and wild populations in this study are all subject to the negative effects of fragmentation. Although *circa situm* and remnant wild populations may conserve adult genetic diversity, the highly correlated paternity of their progenies may compromise the regeneration ability of seeds produced by wild and planted trees alike [83]. These results show that although *circa situm* may be a viable conservation strategy for adult trees, it may limit the regeneration ability of populations in disturbed habitats.

## 5. Conclusions

In summary, we evaluated the differences in genetic diversity and structure and paternity correlation between planted and wild stands of the fruit tree *S*. *purpurea*. Our results indicated that planted trees harbor similar levels of genetic diversity as wild stands. The genetic structure is explained by differences among sites, possibly due to genetic admixture between planted and wild stands within sites, and fruit production is the result of sexual reproduction from pollen flow from a limited number of males and reproductive assurance mechanisms such as apomixis. Therefore, although *circa situm* conditions have maintained moderate levels of genetic diversity in this species, conservation of natural populations may be necessary to increase gene flow and the diversity of pollen donors, which may result in higher fitness of future generations [21, 83]. This in turn, increases the likelihood that populations will persist in the long term. If large undisturbed habitats are not conserved, the genotypes preserved in *circa situm* may only represent a subset of the original genetic diversity of the species and may falsely be considered a genetic reservoir, without considering its relationship with the overall fitness of the species. Therefore, to maximize the effectiveness of *circa situm* strategies in dioecious plants the vegetative propagation of trees should originate from spatially distant individuals to promote high genetic diversity and should strive to maintain equal sex ratios to guarantee sexual reproduction and more diverse progenies in the long term.

## Supporting information

S1 Data (ZIP)

## References

[1] Gentry AH. Patterns of Neotropical Plant Species Diversity. In: Hecht MK, Wallace B, Prance GT, editors. Evolutionary Biology. Boston, MA: Springer US; 1982. pp. 1–84. doi: 10.1007/978-1-4615-6968-8_1

[2] Lott EJ, Bullock SH, Solis-Magallanes JA. Floristic diversity and structure of upland and Arroyo Forests of Coastal Jalisco. Biotropica. 1987;19: 228. doi: 10.2307/2388340

[3] Janzen DH. Tropical Dry Forests: The most endangered major Tropical Ecosystem. In: Peter FM, Wilson EO, editors. Biodiversity. Washington, DC: National Academies Press (US); 1988. pp. 130–137. 25032475

[4] Gillespie TW, Grijalva A, Farris CN. Diversity, composition, and structure of tropical dry forests in Central America. Plant Ecol. 2000;147: 37–47. doi: 10.1023/A:1009848525399

[5] Quesada M, Sanchez-Azofeifa GA, Alvarez-Añorve M, Stoner KE, Avila-Cabadilla L, Calvo-Alvarado J, et al. Succession and management of tropical dry forests in the Americas: Review and new perspectives. For Ecol Manag. 2009;258: 1014–1024. doi: 10.1016/j.foreco.2009.06.023

[6] Bye R. Ethnobotany of the Mexican tropical dry forests. In: Bullock SH, Mooney HA, Medina E, editors. Seasonally Dry Tropical Forests. 1st ed. Cambridge University Press; 1995. pp. 423–438. doi: 10.1017/CBO9780511753398.018

[7] Rosero-Toro JH, Romero-Duque LP, Santos-Fita D, Ruan-Soto F. Cultural significance of the flora of a tropical dry forest in the Doche vereda (Villavieja, Huila, Colombia). J Ethnobiol Ethnomedicine. 2018;14: 22. doi: 10.1186/s13002-018-0220-0 29566709 PMC5865281

[8] Maldonado B, Caballero J, Delgado-Salinas A, Lira R. Relationship between use value and ecological importance of floristic resources of seasonally dry tropical forest in the Balsas iver basin, México. Econ Bot. 2013;67: 17–29. doi: 10.1007/s12231-013-9222-y

[9] Prance GT, Balee W, Boom BM, Carneiro RL. Quantitative ethnobotany and the case for conservation in *Ammonia*. Conserv Biol. 1987;1: 296–310. doi: 10.1111/j.1523-1739.1987.tb00050.x

[10] Peters CM, Balick MJ, Kahn F, Anderson AB. Oligarchic forests of economic plants in Amazonia: Utilization and conservation of an important tropical resource. Conserv Biol. 1989;3: 341–349. doi: 10.1111/j.1523-1739.1989.tb00240.x 21129021

[11] Prance GT. Indigenous non-timber benefits from tropical rain forest. In: Goldsmith FB, editor. Tropical Rain Forest: A Wider Perspective. Dordrecht: Springer Netherlands; 1998. pp. 21–42. doi: 10.1007/978-94-011-4912-9_2

[12] Murphy PG, Lugo AE. Ecology of Tropical Dry Forest. Annu Rev Ecol Syst. 1986;17: 67–88. doi: 10.1146/annurev.es.17.110186.000435

[13] DRYFLOR Banda-RK, Delgado-Salinas A, Dexter KG, Linares-Palomino R, Oliveira-Filho A, et al. Plant diversity patterns in neotropical dry forests and their conservation implications. Science. 2016;353: 1383–1387. doi: 10.1126/science.aaf5080 27708031

[14] Miles L, Newton AC, DeFries RS, Ravilious C, May I, Blyth S, et al. A global overview of the conservation status of tropical dry forests. J Biogeogr. 2006;33: 491–505. doi: 10.1111/j.1365-2699.2005.01424.x

[15] Murphy PG, Lugo AE. Dry forests of Central America and the Caribbean. 1st ed. In: Bullock SH, Mooney HA, Medina E, editors. Seasonally Dry Tropical Forests. Cambridge University Press; 1995. pp. 9–34. doi: 10.1017/CBO9780511753398.002

[16] Boshier D, Gordon J, Barrance A. Prospects for *Circa Situm* tree conservation in Mesoamerican Dry-Forest Agro-ecosystems. Biodiversity Conservation in Costa Rica: Learning the Lessons in a Seasonal Dry Forest. 2004. pp. 210–226. doi: 10.1525/california/9780520223097.003.0016

[17] Quesada M, Stoner KE. Threats to the conservation of tropical dry forest in Costa Rica. In: Frankie G, Mata A, Vinson SB, editors. Biodiversity conservation in Costa Rica: Learning the lessons in a Seasonal Dry Forest. University of California Press; 2004. doi: 10.1525/california/9780520223097.001.0001

[18] Aguilar R, Ashworth L, Galetto L, Aizen MA. Plant reproductive susceptibility to habitat fragmentation: review and synthesis through a meta-analysis. Ecol Lett. 2006;9: 968–980. doi: 10.1111/j.1461-0248.2006.00927.x 16913941

[19] Aguilar R, Quesada M, Ashworth L, Herrerias-Diego Y, Lobo J. Genetic consequences of habitat fragmentation in plant populations: susceptible signals in plant traits and methodological approaches. Mol Ecol. 2008;17: 5177–5188. doi: 10.1111/j.1365-294X.2008.03971.x 19120995

[20] Fuchs EJ, Lobo JA, Quesada M. Effects of forest fragmentation and flowering phenology on the reproductive success and mating patterns of the tropical dry forest tree *Pachira quinata*. Conserv Biol. 2003;17: 149–157. doi: 10.1046/j.1523-1739.2003.01140.x

[21] Cascante A, Quesada M, Lobo JJ, Fuchs EA. Effects of dry tropical forest fragmentation on the reproductive success and genetic structure of the tree *Samanea saman*. Conserv Biol. 2002;16: 137–147. doi: 10.1046/j.1523-1739.2002.00317.x 35701973

[22] Cristóbal-Pérez EJ, Fuchs EJ, Martén-Rodríguez S, Quesada M. Habitat fragmentation negatively affects effective gene flow via pollen, and male and female fitness in the dioecious tree, *Spondias purpurea* (Anacardiaceae). Biol Conserv. 2021;256: 109007. doi: 10.1016/j.biocon.2021.109007

[23] Fuchs EJ, Hamrick JL. Mating system and pollen flow between remnant populations of the endangered tropical tree, *Guaiacum sanctum* (Zygophyllaceae). Conserv Genet. 2011;12: 175–185. doi: 10.1007/s10592-010-0130-8

[24] Aldrich PR, Hamrick JL. Reproductive dominance of pasture trees in a fragmented tropical forest mosaic. Science. 1998;281: 103–105. doi: 10.1126/science.281.5373.103 9651242

[25] Ricketts TH, Daily GC, Ehrlich PR, Michener CD. Economic value of tropical forest to coffee production. Proc Natl Acad Sci. 2004;101: 12579–12582. doi: 10.1073/pnas.0405147101 15306689 PMC515099

[26] Harvey CA, González Villalobos JA. Agroforestry systems conserve species-rich but modified assemblages of tropical birds and bats. Biodivers Conserv. 2007;16: 2257–2292. doi: 10.1007/s10531-007-9194-2

[27] Tscharntke T, Sekercioglu CH, Dietsch TV, Sodhi NS, Hoehn P, Tylianakis JM. Landscape constraints on functional diversity of birds and insects in tropical agroecosystems. Ecology. 2008;89: 944–951. doi: 10.1890/07-0455.1 18481519

[28] Dawson IK, Guariguata MR, Loo J, Weber JC, Lengkeek A, Bush D, et al. What is the relevance of smallholders’ agroforestry systems for conserving tropical tree species and genetic diversity in circa situm, in situ and ex situ settings? A review. Biodivers Conserv. 2013;22: 301–324. doi: 10.1007/s10531-012-0429-5

[29] Han L, Love K, Peace B, Broadhurst L, England N, Li L, et al. Origin of planted Eucalyptus benthamii trees in Camden NSW: checking the effectiveness of *Circa Situm* conservation measures using molecular markers. Biodivers Conserv. 2020;29: 1301–1322. doi: 10.1007/s10531-020-01936-4

[30] Novello M, Viana JPG, Alves-Pereira A, de Aguiar Silvestre E, Nunes HF, Pinheiro JB, et al. Genetic conservation of a threatened Neotropical palm through community-management of fruits in agroforests and second-growth forests. For Ecol Manag. 2018;407: 200–209. doi: 10.1016/j.foreco.2017.06.059

[31] Reis MS, Montagna T, Mattos AG, Filippon S, Ladio AH, Marques A da C, et al. Domesticated landscapes in Araucaria Forests, Southern Brazil: a multispecies local conservation-by-use system. Front Ecol Evol. 2018;6: 11. doi: 10.3389/fevo.2018.00011

[32] Donazzolo J, Stefenon VM, Guerra MP, Nodari RO. On farm management of *Acca sellowiana* (Myrtaceae) as a strategy for conservation of species genetic diversity. Sci Hortic. 2020;259: 108826. doi: 10.1016/j.scienta.2019.108826

[33] de Souza Milanesi L, Montagna T, dos Reis MS, Peroni N. Population Biology of Palm Heart (Euterpe edulis Martius–Arecaceae) in Managed Landscape Units in Southern Brazil. Econ Bot. 2021;75: 144–157. doi: 10.1007/s12231-021-09519-2

[34] Miller AJ, Schaal BA. Domestication and the distribution of genetic variation in wild and cultivated populations of the Mesoamerican fruit tree *Spondias purpurea* L. (Anacardiaceae). Mol Ecol. 2006;15: 1467–1480. doi: 10.1111/j.1365-294X.2006.02834.x 16629804

[35] Miller A, Schaal B. Domestication of a mesoamerican cultivated fruit tree, *Spondias purpurea*. Proc Natl Acad Sci USA. 2005;102: 12801–12806. doi: 10.1073/pnas.0505447102 16126899 PMC1200284

[36] Miller AJ, Knouft JH. GIS-based characterization of the geographic distributions of wild and cultivated populations of the Mesoamerican fruit tree Spondias purpurea (Anacardiaceae). Am J Bot. 2006;93: 1757–1767. doi: 10.3732/ajb.93.12.1757 21642121

[37] Miller AJ, Gross BL. From forest to field: Perennial fruit crop domestication. Am J Bot. 2011;98: 1389–1414. doi: 10.3732/ajb.1000522 21865506

[38] Cristóbal-Pérez EJ, Fuchs EJ, Olivares-Pinto U, Quesada M. Janzen-Connell effects shape gene flow patterns and realized fitness in the tropical dioecious tree *Spondias purpurea* (Anacardiaceae). Sci Rep. 2020;10: 4584. doi: 10.1038/s41598-020-61394-4 32165645 PMC7067871

[39] Werden LK, Alvarado JP, Zarges S, Calderón ME, Schilling EM, Gutiérrez L. M, et al. Using soil amendments and plant functional traits to select native tropical dry forest species for the restoration of degraded Vertisols. Nuñez M, editor. J Appl Ecol. 2018;55: 1019–1028. doi: 10.1111/1365-2664.12998

[40] Waring BG, Pérez-Aviles D, Murray JG, Powers JS. Plant community responses to stand-level nutrient fertilization in a secondary tropical dry forest. Ecology. 2019;100. doi: 10.1002/ecy.2691 30989648

[41] Wilson K, Hardy ICW. Statistical analysis of sex ratios: an introduction. In: Hardy ICW, editor. Sex ratios: Concepts and research methods. Cambridge: Cambridge University Press; 2002. pp. 48–92. doi: 10.1017/CBO9780511542053.004

[42] R Core Team. R: A language and environment for statistical. Vienna, Austria.: 2022. https://www.R-project.org/.

[43] Doyle JJ, Doyle JL. A rapid DNA isolation procedure for small quantities of fresh leaf tissue. Phytochem Bull. 1987;19: 11–15.

[44] Cristóbal-Pérez EJ, Fuchs EJ, Harvey N, Quesada M. Isolation and characterization of microsatellites loci in Spondias purpurea (Anacardiaceae) and cross amplification in congeneric species. Mol Biol Rep. 2019;46: 5581–5585. doi: 10.1007/s11033-019-04968-4 31321644

[45] Van Oosterhout C, Hutchinson WF, Wills DPM, Shipley P. MICRO-CHECKER: software for identifying and correcting genotyping errors in microsatellite data. Mol Ecol Notes. 2004;4: 535–538. doi: 10.1111/j.1471-8286.2004.00684.x

[46] Goudet J. hierfstat, a package for r to compute and test hierarchical F-statistics. Mol Ecol Notes. 2005;5: 184–186. doi: 10.1111/j.1471-8286.2004.00828.x

[47] Kamvar ZN, Tabima JF, Grünwald NJ. Poppr: an R package for genetic analysis of populations with clonal, partially clonal, and/or sexual reproduction. PeerJ. 2014;2: e281. doi: 10.7717/peerj.281 24688859 PMC3961149

[48] Meirmans PG, Van Tienderen PH. genotype and genodive: two programs for the analysis of genetic diversity of asexual organisms. Mol Ecol Notes. 2004;4: 792–794. doi: 10.1111/j.1471-8286.2004.00770.x

[49] Peakall R, Smouse PE. GenAlEex 6: genetic analysis in Excel. Population genetic software for teaching and research. Mol Ecol Notes. 2006;6: 288–295. doi: 10.1111/j.1471-8286.2005.01155.x PMC3463245 22820204

[50] Peakall R, Smouse PE. GenAlEx 6.5: genetic analysis in Excel. Population genetic software for teaching and research-an update. Bioinformatics. 2012;28: 2537–2539. doi: 10.1093/bioinformatics/bts460 22820204 PMC3463245

[51] Dorken ME, Eckert CG. Severely reduced sexual reproduction in northern populations of a clonal plant, *Decodonverticillatus* (Lythraceae). J Ecol. 2001;89: 339–350. doi: 10.1046/j.1365-2745.2001.00558.x

[52] Pritchard JK, Stephens M, Donnelly P. Inference of population structure using multilocus genotype data. Genetics. 2000;155: 945–959. doi: 10.1093/genetics/155.2.945 10835412 PMC1461096

[53] Falush D, Stephens M, Pritchard JK. Inference of population structure using multilocus genotype data: linked loci and correlated allele frequencies. Genetics. 2003;164: 1567–1587. doi: 10.1093/genetics/164.4.1567 12930761 PMC1462648

[54] Earl DA, vonHoldt BM. STRUCTURE HARVESTER: a website and program for visualizing STRUCTURE output and implementing the Evanno method. Conserv Genet Resour. 2012;4: 359–361. doi: 10.1007/s12686-011-9548-7

[55] Jakobsson M, Rosenberg NA. CLUMPP: a cluster matching and permutation program for dealing with label switching and multimodality in analysis of population structure. Bioinformatics. 2007;23: 1801–1806. doi: 10.1093/bioinformatics/btm233 17485429

[56] Francis RM. pophelper: an R package and web app to analyse and visualize population structure. Mol Ecol Resour. 2017;17: 27–32. doi: 10.1111/1755-0998.12509 26850166

[57] Nei M. Molecular Evolutionary Genetics. New York, NY: Columbia University Press; 1987.

[58] Loiselle BA, Sork VL, Nason J, Graham C. Spatial genetic structure of a tropical understory shrub, *Psychotria officinalis* (Rubiaceae). Am J Bot. 1995;82: 1420–1425. doi: 10.2307/2445869

[59] Ritland K. Extensions of models for the estimation of mating systems using n independent loci. Heredity. 2002;88: 221–228. doi: 10.1038/sj.hdy.6800029 11920127

[60] Hamrick JL. Response of forest trees to global environmental changes. For Ecol Manag. 2004;197: 323–335. doi: 10.1016/j.foreco.2004.05.023

[61] Dick CW, Hardy OJ, Jones FA, Petit RJ. Spatial scales of pollen and seed-mediated gene flow in tropical rain forest trees. Trop Plant Biol. 2008;1: 20–33. doi: 10.1007/s12042-007-9006-6

[62] Riba-Hernández P, Segura JL, Fuchs EJ, Moreira J. Population and genetic structure of two dioecious timber species *Virola surinamensis* and *Virola koschnyi* (Myristicaceae) in southwestern Costa Rica. For Ecol Manag. 2014;323: 168–176. doi: 10.1016/j.foreco.2014.03.018

[63] Silva BM, Rossi AAB, Tiago AV, Schmitt KFM, Dardengo JFE, Souza SAM. Genetic diversity of Cajazeira (*Spondias mombin* L.) in three geographic regions. Genet Mol Res. 2017;16. doi: 10.4238/gmr16018946 28128399

[64] Santos VN dos, Fernandes Santos CA, Ribeiro de Oliveira V, Da Silva Costa AE, Santos da Silva FF. Diversity and genetic structure of Spondias tuberosa (Anacardiaceae) accessions based on microsatellite loci. Rev Biol Trop. 2021;69: 640–648. doi: 10.15517/rbt.v69i2.44194

[65] Müller Zortéa KÉ, Bandini Rossi AA, Vicente Tiago A, Dos Santos Cardoso E, Andrade Pinto JM, Sobreira Hoogerheid ES. The *Spondias mombin* (Anarcadiaceae): molecular characterization and conservation. Rev Biol Trop. 2021;69: 1023–1036. doi: 10.15517/rbt.v69i3.45810

[66] Ross-Ibarra J, Morrell PL, Gaut BS. Plant domestication, a unique opportunity to identify the genetic basis of adaptation. Proc Natl Acad Sci. 2007;104: 8641–8648. doi: 10.1073/pnas.0700643104 17494757 PMC1876441

[67] Nassar NMA, Ortiz R. Cassava improvement: challenges and impacts. J Agric Sci. 2007;145: 163. doi: 10.1017/S0021859606006575

[68] Malik AA, Singh VK, Sharma SS, Negi MS, Tripathi SB. Prevalence of apomixis in *Jatropha*: Is it significant? Biofuels. 2022;13: 365–369. doi: 10.1080/17597269.2019.1706280

[69] Martins-Lopes P, Gomes S, Lima-Brito J, Lopes J, Guedes-Pinto H. Assessment of clonal genetic variability in *Olea europaea* L. ‘Cobrançosa’ by molecular markers. Sci Hortic. 2009;123: 82–89. doi: 10.1016/j.scienta.2009.08.001

[70] Achtak H, Ater M, Oukabli A, Santoni S, Kjellberg F, Khadari B. Traditional agroecosystems as conservatories and incubators of cultivated plant varietal diversity: the case of fig (*Ficus carica* L.) in Morocco. BMC Plant Biol. 2010;10: 28. doi: 10.1186/1471-2229-10-28 20167055 PMC2844065

[71] Scarcelli N, Couderc M, Baco MN, Egah J, Vigouroux Y. Clonal diversity and estimation of relative clone age: application to agrobiodiversity of yam (*Dioscorea rotundata*). BMC Plant Biol. 2013;13: 178. doi: 10.1186/1471-2229-13-178 24219837 PMC3832681

[72] Chumacero de Schawe C, Durka W, Tscharntke T, Hensen I, Kessler M. Gene flow and genetic diversity in cultivated and wild cacao (*Theobroma cacao*) in Bolivia. Am J Bot. 2013;100: 2271–2279. doi: 10.3732/ajb.1300025 24158148

[73] Chiapero AL, Aguilar R, Galfrascoli GM, Bernardello G, Quesada M, Ashworth L. Reproductive resilience to habitat fragmentation of *Lithraea molleoides* (Anacardiaceae), a dominant dioecious tree from the Chaco Serrano. For Ecol Manag. 2021;492: 119215. doi: 10.1016/j.foreco.2021.119215

[74] Richards AJ. Apomixis in flowering plants: an overview. Philos Trans R Soc B Biol Sci. 2003;358: 1085–1093. doi: 10.1098/rstb.2003.1294 12831474 PMC1693200

[75] Hojsgaard D, Klatt S, Baier R, Carman JG, Hörandl E. Taxonomy and biogeography of apomixis in angiosperms and associated biodiversity characteristics. Crit Rev Plant Sci. 2014;33: 414–427. doi: 10.1080/07352689.2014.898488 27019547 PMC4786830

[76] Ellstrand NC, Prentice HC, Hancock JF. Gene flow and introgression from domesticated plants into their wild relatives. Annu Rev Ecol Syst. 1999;30: 539–563. doi: 10.1146/annurev.ecolsys.30.1.539

[77] Hulshof CM, Powers JS. Tropical forest composition and function across space and time: Insights from diverse gradients in Área de Conservación Guanacaste. Biotropica. 2020;52: 1065–1075. doi: 10.1111/btp.12689

[78] Bawa KS. Evolution of dioecy in flowering plants. Annu Rev Ecol Syst. 1980;11: 15–39. doi: 10.1146/annurev.es.11.110180.000311

[79] Ashman T-L, Knight TM, Steets JA, Amarasekare P, Burd M, Campbell DR, et al. Pollen limitation of plant reproduction: Ecological and evolutionary causes and consequences. Ecology. 2004;85: 2408–2421. doi: 10.1890/03-8024

[80] Knight TM, Steets JA, Vamosi JC, Mazer SJ, Burd M, Campbell DR, et al. Pollen Limitation of Plant Reproduction: Pattern and Process. Annu Rev Ecol Evol Syst. 2005;36: 467–497. doi: 10.1146/annurev.ecolsys.36.102403.115320

[81] Bianchi FJJA Cunningham SA. Unravelling the role of mate density and sex ratio in competition for pollen. Oikos. 2012;121: 219–227. doi: 10.1111/j.1600-0706.2011.19842.x

[82] Karron JD, Mitchell RJ, Holmquist KG, Bell JM, Funk B. The influence of floral display size on selfing rates in *Mimulus ringens*. Heredity. 2003;92: 242–248. doi: 10.1038/sj.hdy.6800402 14666135

[83] Aguilar R, Cristóbal-Pérez EJ, Balvino-Olvera FJ, Aguilar-Aguilar M, Aguirre-Acosta N, Ashworth L, et al. Habitat fragmentation reduces plant progeny quality: a global synthesis. Gomez JM, editor. Ecol Lett. 2019;22: 1163–1173. doi: 10.1111/ele.13272 31087604

[84] Karron JD, Mitchell RJ. Effects of floral display size on male and female reproductive success in *Mimulus ringens*. Ann Bot. 2012;109: 563–570. doi: 10.1093/aob/mcr193 21880660 PMC3278290

