# Supplementary material for: Genetic diversity, asexual reproduction and conservation of the edible fruit tree Spondias purpurea L. (Anacardiaceae) in the Costa Rican tropical dry forest
Source: PLoS One. 2022 Nov 17;17(11):e0277439. doi: 10.1371/journal.pone.0277439 (PMC9671346; doi:10.1371/journal.pone.0277439)
Supplement: S1 Data — (ZIP) [file pone.0277439.s001.zip › Supporting Information/S1 TABLE.docx]

| Group | Locality and size class | N | N_a_ | ***A_r_*** | H_o_ | H_e_ | F |
| --- | --- | --- | --- | --- | --- | --- | --- |
| Planted | AC - Adults | 22 | 3.86  (0.705) | 2.98  (0.307) | 0.642  (0.139) | 0.50  (0.215) | -0.208  (0.19) |
|  | AC - Seeds | 141 | 4.86  (0.829) | 1.932  (0.220) | 0.47  (0.108) | 0.437  (0.069) | -0.063  (0.15) |
|  | MU - Adults | 16 | 3  (0.436) | 2.20  (0.401) | 0.49  (0.130) | 0.46  (0.089) | 0.04  (0.21) |
|  | MU - Seeds | 154 | 3.58  (0.429) | 1.93  (0.149) | 0.75  (0.124) | 0.46  (0.048) | -0.55  (0.154) |
|  | HO - Adults | 8 | 3.15  (0.34) | 2.37  (0.292) | 0.50  (0.093) | 0.57  (0.063) | 0.08  (0.132) |
|  | HO - Seeds | 116 | 3.45  (0.481) | 2.36  (0.268) | 0.52  (0.121) | 0.53  (0.077) | -0.04  (0.206) |
|  | **Mean** |  | **23.3** | **2.92** | **0.54** | **0.51** | **0.13** |
| Wild | AC- Adults | 29 | 4  (0.535) | 2.3  (0.238) | 0.65  (0.141) | 0.51  (0.074) | -0.192  (0.198) |
|  | AC - Seeds | 84 | 4.86  (0.829) | 1.94  (0.22) | 0.47  (0.108) | 0.44  (0.069) | -0.063  (0.15) |
|  | MU - Adults | 25 | 3.6  (0.459) | 3.58  (0.369) | 0.55  (0.071) | 0.55  (0.046) | -0.01  (0.122) |
|  | MU - Seeds | 47 | 3.72  (0.522) | 2.32  (0.298) | 0.66  (0.106) | 0.53  (0.053) | -0.22  (0.159) |
|  | HO - Adults | 101 | 5  (0.787) | 1.87  (0.22) | 0.34  (0.127) | 0.40  (0.084) | 0.24  (0.217) |
|  | HO- Seeds | 106 | 3.8  (0.522) | 2.32  (0.298) | 0.66  (0.106) | (0.53)  (0.053) | -0.22  (0.159) |
|  | **Mean** |  | **29.3** | **2.85** | **0.50** | **0.49** | **0.14** |
|  | **Grand mean** |  | **3.85**  **(0.18)** | **2.17**  **(0.077)** | **0.55**  **(0.034)** | **0.49**  **(0.02)** | **-0.01**  **(0.052)** |
