# Supplementary material for: Genetic diversity, asexual reproduction and conservation of the edible fruit tree Spondias purpurea L. (Anacardiaceae) in the Costa Rican tropical dry forest
Source: PLoS One. 2022 Nov 17;17(11):e0277439. doi: 10.1371/journal.pone.0277439 (PMC9671346; doi:10.1371/journal.pone.0277439)
Supplement: S1 Data — (ZIP) [file pone.0277439.s001.zip › Supporting Information/S2 TABLE.docx]

| Group | Size Class | N | N_a_ | ***A_r_*** | H_o_ | H_e_ | F |
| --- | --- | --- | --- | --- | --- | --- | --- |
| Planted | Adults | 21 | 3.095  (0.208) | 2.426  (0.136) | 0.519  (0.055) | 0.524  (0.023) | 0.031  (0.068) |
|  | Seeds | 102 | 3.810  (0.172) | 2.269  (0.134) | 0.552  (0.067) | 0.503  (0.032) | -0.119  (0.062) |
|  | **Mean** |  | **3.476** | **2.359** | **0.538** | **0.519** | **-0.039** |
| Wild | Adults | 92 | 3.095  (0.208) | 2.426  (0.136) | 0.519  (0.055) | 0.524  (0.023) | 0.031  (0.068) |
|  | Seeds | 147 | 3.857  (0.502) | 2.292  (0.042) | 0.557  (0.080) | 0.514  (0.010) | -0.109  (0.119) |
|  | **Mean** |  | **3.905** | **2.296** | **0.540** | **0.507** | **-0.056** |
