# Supplementary material for: Genetic diversity, asexual reproduction and conservation of the edible fruit tree Spondias purpurea L. (Anacardiaceae) in the Costa Rican tropical dry forest
Source: PLoS One. 2022 Nov 17;17(11):e0277439. doi: 10.1371/journal.pone.0277439 (PMC9671346; doi:10.1371/journal.pone.0277439)
Supplement: S1 Data — (ZIP) [file pone.0277439.s001.zip › Supporting Information/S3 TABLE.docx]

| Statistics | Planted | Wild | OSx | p-value |
| --- | --- | --- | --- | --- |
| H_E_ | 0.516 | 0.496 | 0.020 | 0.679 |
| H_O_ | 0.545 | 0.512 | 0.032 | 0.901 |
| F_IS_ | -0.056 | -0.033 | 0.023 | 0.901 |
| G_ST_ | 0.052 | 0.154 | 0.102 | 0.682 |
