# Supplementary material for: Genetic diversity, asexual reproduction and conservation of the edible fruit tree Spondias purpurea L. (Anacardiaceae) in the Costa Rican tropical dry forest
Source: PLoS One. 2022 Nov 17;17(11):e0277439. doi: 10.1371/journal.pone.0277439 (PMC9671346; doi:10.1371/journal.pone.0277439)
Supplement: S1 Data — (ZIP) [file pone.0277439.s001.zip › Supporting Information/S4 TABLE.docx]

| **Study site** | **Males** | **Females** | **Sex ratio** |
| --- | --- | --- | --- |
| AC | 14 | 39 | 0.26 ^*^ |
| MU | 9 | 40 | 0.22 ^*^ |
| HO | 64 | 68 | 0.48 ^NS^ |
| **Grand Total** | **92** | **147** | **0.47 ^NS^** |

NS: Nonsignificant deviations from 1:1 sex ratio (p>0.05).

* : indicates significant deviations from 1:1 sex ratio (p<0.01)
