# Supplementary material for: Genetic diversity, asexual reproduction and conservation of the edible fruit tree Spondias purpurea L. (Anacardiaceae) in the Costa Rican tropical dry forest
Source: PLoS One. 2022 Nov 17;17(11):e0277439. doi: 10.1371/journal.pone.0277439 (PMC9671346; doi:10.1371/journal.pone.0277439)
Supplement: S1 Data — (ZIP) [file pone.0277439.s001.zip › Supporting Information/S5 TABLE.docx]

|  |  | **Planted** | | | **Wild** | | |
| --- | --- | --- | --- | --- | --- | --- | --- |
|  |  | **AC** | **MU** | **HO** | **AC** | **MU** | **HO** |
| **Planted** | **AC** |  | 0.111 | **0.069** | -0.005 | **0.083** | **0.318** |
|  | **MU** |  |  | 0.047 | **0.121** | 0.035 | **0.358** |
|  | **HO** |  |  |  | **0.078** | 0.005 | **0.227** |
| **Wild** | **AC** |  |  |  |  | **0.096** | **0.325** |
|  | **MU** |  |  |  |  |  | **0.207** |
|  | **HO** |  |  |  |  |  |  |
