# Supplementary material for: Genetic diversity, asexual reproduction and conservation of the edible fruit tree Spondias purpurea L. (Anacardiaceae) in the Costa Rican tropical dry forest
Source: PLoS One. 2022 Nov 17;17(11):e0277439. doi: 10.1371/journal.pone.0277439 (PMC9671346; doi:10.1371/journal.pone.0277439)
Supplement: S1 Data — (ZIP) [file pone.0277439.s001.zip › Supporting Information/S6 TABLE.docx]

|  |  | **Planted** | | | **Wild** | | |
| --- | --- | --- | --- | --- | --- | --- | --- |
|  |  | **AC** | **MU** | **HO** | **AC** | **MU** | **HO** |
| **Planted** | **AC** |  | 0.074 | 0.029 | -0.006 | **0.056** | **0.188** |
|  | **MU** |  |  | 0.004 | **0.072** | -0.015 | **0.238** |
|  | **HO** |  |  |  | **0.052** | 0.007 | **0.189** |
| **Wild** | **AC** |  |  |  |  | **0.075** | **0.263** |
|  | **MU** |  |  |  |  |  | **0.192** |
|  | **HO** |  |  |  |  |  |  |
