# Supplementary material for: Genetic diversity, asexual reproduction and conservation of the edible fruit tree Spondias purpurea L. (Anacardiaceae) in the Costa Rican tropical dry forest
Source: PLoS One. 2022 Nov 17;17(11):e0277439. doi: 10.1371/journal.pone.0277439 (PMC9671346; doi:10.1371/journal.pone.0277439)
Supplement: S1 Data — (ZIP) [file pone.0277439.s001.zip › Supporting Information/S7 TABLE.docx]

|  | **PAC** | **PMU** | **PHO** | **WAC** | **WMU** | **WHO** |
| --- | --- | --- | --- | --- | --- | --- |
| **PAC** | 0.271 | 0.199 | 0.135 | 0.263 | 0.136 | 0.02 |
| **PMU** |  | 0.331 | 0.185 | 0.188 | 0.211 | 0.024 |
| **PHO** |  |  | 0.193 | 0.132 | 0.131 | 0.025 |
| **WAC** |  |  |  | 0.274 | 0.125 | 0.012 |
| **WMU** |  |  |  |  | 0.172 | 0.044 |
| **WHO** |  |  |  |  |  | 0.18 |
